# Unravelling IgG4-Related Disease: A Case of Systemic Inflammation and Autoimmune Overlap

**DOI:** 10.7759/cureus.117463

**Published:** 2026-10-05

**Authors:** Radwa Atef, Ayesha Banu, Mohammad Kais A Jawish, Raabeah F Jafri, Namra F Jafri, Yazan Y Rabah, Leena Abdelrahman

**Affiliations:** 1 Internal Medicine, Dubai Health Authority, Dubai, ARE; 2 Medicine, Dubai Health, Dubai, ARE; 3 Department of Clinical Sciences, College of Medicine, University of Sharjah, Sharjah, ARE; 4 Department of Clinical Sciences, Dubai Medical College for Girls, Dubai, ARE; 5 Internal Medicine, Dubai Hospital, Dubai Health, Dubai, ARE

**Keywords:** epstein-barr virus, igg4-related disease, polyclonal hypergammaglobulinemia, rare cause of diffuse lymphadenopathy, storiform fibrosis

## Abstract

Immunoglobulin G4-related disease (IgG4-RD) is a rare, chronic, immune-mediated disorder characterized by fibroinflammatory changes and infiltration of IgG4-positive plasma cells; its clinical spectrum overlaps with malignancies, infections, and autoimmune conditions, often complicating diagnosis. We report a 47-year-old woman with a history of seronegative rheumatoid arthritis, antiphospholipid syndrome, and diabetes mellitus who presented with low-grade fever, left flank pain, and generalized lymphadenopathy. Laboratory findings revealed markedly elevated CRP and IgG4, and imaging demonstrated pulmonary infiltrates and widespread lymphadenopathy concerning for malignancy. Excisional lymph node biopsy showed plasma cell-rich hyperplasia with increased IgG4-positive plasma cells, and Epstein-Barr virus (EBV) and cytomegalovirus (CMV) serologies suggested recent or reactivated infection. The patient was diagnosed with IgG4-RD and treated with corticosteroids followed by rituximab, resulting in clinical improvement. This case illustrates the diagnostic complexity of IgG4-RD in the presence of systemic inflammation, autoimmune background, and viral reactivation; its pathogenesis involves atypical immune regulation between B cells and CD4+ cytotoxic T cells that drives cytokine-mediated fibroblast activation, leading to storiform fibrosis and lymphoplasmacytic infiltration. EBV infection may have acted as a trigger for IgG4 class switching, though its role remains uncertain, and early histopathological confirmation with prompt treatment is critical to prevent irreversible organ damage. IgG4-RD should be considered in patients with unexplained systemic lymphadenopathy and elevated inflammatory markers after exclusion of infection and malignancy; a multidisciplinary approach is essential for diagnosis and management, and this case underscores the potential contribution of viral triggers, particularly EBV, warranting further research.

## Introduction

IgG4 is the least common subtype of immunoglobulins. Due to its unique structure, IgG4 is regarded as a non-inflammatory, tolerance-inducing immunoglobulin [1]. Immunoglobulin G4-related disease (IgG4-RD) is a chronic, immune-mediated, multisystem condition characterized by significant fibroinflammatory changes [2]. Although any organ system can be affected, the pancreas, salivary glands, lacrimal glands, bile ducts, retroperitoneum, aorta, and thyroid are among the most commonly involved sites. The exact etiology remains unclear, but it is believed to involve genetic and autoimmune components [3]. The global prevalence of the disease remains unclear due to limited epidemiological data. A study conducted in the Ishikawa prefecture, Japan (population of 1.14 million) estimated an annual incidence of 0.28-1.08 per 100,000 individuals, with a mean age at diagnosis of 58 years. Although IgG4-RD most commonly affects individuals over the age of 50, it has also been documented in younger adults and, rarely, in children [4].

Clinically, patients often present with tumor-like swellings or masses in the affected organs [5]. Constitutional symptoms of IgG4-RD, such as fever or weight loss, are rare due to the indolent nature of the disease. Common organ-specific symptoms may include sicca syndrome with bilateral parotid and sublingual gland enlargement. Involvement of the lungs often manifests similarly to malignancy, with shortness of breath, chronic cough, and recurrent pleural effusions. In the retroperitoneum, the disease can manifest as retroperitoneal fibrosis, leading to lower back pain, deep vein thrombosis, and lower limb oedema [6].

Diagnosis relies on the combination of clinical features along with three main components: radiological findings, serological tests, and, most significantly, histopathological findings. Recent revisions to diagnostic guidelines have added storiform fibrosis and obliterative phlebitis as significant pathological features [7]. An elevated serum IgG4 level is also a key diagnostic marker and is included in many diagnostic criteria for the disease [8]. A notable and clinically important feature of IgG4-RD is its typically excellent response to corticosteroid therapy, making early recognition and treatment critical to prevent irreversible organ damage [9]. In this case report, we present a 47-year-old woman with a confirmed diagnosis of IgG4-RD, showcasing her atypical presentation, diagnostic course, and clinical outcomes.

## Case presentation

A 47-year-old woman, a known case of seronegative rheumatoid arthritis on leflunomide, antiphospholipid syndrome on warfarin, insulin-dependent diabetes mellitus, peripheral vascular disease, and a prior left below-knee amputation due to gangrenous diabetic complications, presented to the emergency department with a persistent, low-grade intermittent fever for the last three months, accompanied by left flank pain and constipation. There were no night sweats, significant weight loss, nausea, vomiting, or abdominal distension. She denied sicca symptoms suggestive of sicca syndrome, including dry eyes, dry mouth, or salivary gland swelling.

On presentation, she appeared febrile and mildly pale, with localized tenderness over the left flank. She was vitally stable. Laboratory investigations demonstrated a markedly elevated CRP of 222 mg/L. Urinalysis revealed evidence of a urinary tract infection (UTI); however, both urine and blood cultures were negative. In the absence of microbiological confirmation and a clinical course inconsistent with infection, UTI was considered unlikely to fully explain the patient's systemic symptoms.

Other labs obtained as part of her workup included human herpesvirus 8 (HHV8) and cytomegalovirus (CMV), which were negative in the tissue. However, CMV and Epstein-Barr virus (EBV) serologies were positive for both IgG and IgM, suggesting recent or reactivated infection. EBV DNA PCR was also positive but below the quantification threshold. ANA was positive at 1:100 (undefined pattern); further ANA profiling was not pursued, as it was regarded as a nonspecific finding in the absence of clinical features suggestive of a defined connective tissue disease. Direct antiglobulin test (DAT) revealed anti-C3d negativity with anti-IgG positivity, consistent with autoimmune hemolysis. The patient also had elevated immunoglobulin G subclasses, including IgG4, as shown in Table 1. 

**Table 1:** Laboratory findings on admission. IFA: immunofluorescence assay, EBV: Epstein-Barr virus, VCA: viral capsid antigen, IgG: immunoglobulin G, IgM: immunoglobulin M, EA: early antigen, EBNA: Epstein-Barr nuclear antigen, DNA: deoxyribonucleic acid, PCR: polymerase chain reaction, CMV: cytomegalovirus, HHV8: human herpesvirus 8, IgG4-RD: immunoglobulin G subclass 4-related disease

Test	Result	Reference Range	Interpretation
C-Reactive Protein (CRP)	222 mg/L	<5 mg/L	Markedly elevated, indicating systemic inflammation
Antinuclear Antibody (ANA) by IFA	Positive 1:100, undefined pattern	Negative; <1:100	Weakly positive; nonspecific in the absence of a defined connective tissue disease
EBV Antibody Profile (Blood, Venous)			
EBV VCA IgG	Positive	Negative	Suggests past or recent exposure
EBV VCA IgM	Positive	Negative	Suggests recent/reactivated infection; later deemed a false positive
EBV EA IgG	Positive	Negative	Suggests recent viral replication
EBV EBNA IgG	Positive	Negative	Indicates past infection
EBV DNA PCR (Blood)	Positive, below quantification threshold	Negative	Detectable but not a clinically significant viremia
Cytomegalovirus Antibody (Blood, Venous)			
Cytomegalovirus IgG	Positive	Negative	Past exposure
Cytomegalovirus IgM	Positive	Negative	Suggests recent/reactivated infection; later deemed a false positive
Fibrinogen	725.34 mg/dL	200-400 mg/dL	Elevated, consistent with an acute-phase response
IL-6 (Interleukin 6)	394.00 pg/mL	<7.0 pg/mL	Markedly elevated, reflecting systemic inflammatory cytokine activity
Direct Antiglobulin Test (DAT), Monospecific (IgG + C3)			
DAT Anti-IgG	Positive	Negative	Consistent with warm autoimmune hemolysis
DAT Anti-C3d	Negative	Negative	Argues against complement-mediated hemolysis
Tissue Studies (Lymph Node)			
HHV8 (immunohistochemistry/in situ hybridization)	Negative	Negative	Argues against HHV8-associated multicentric Castleman disease
CMV (tissue)	Negative	Negative	Argues against active tissue CMV infection
Immunoglobulin G Subclasses			
Immunoglobulin G1 (IgG1)	11.97 g/L	3.824-9.286 g/L	Elevated
Immunoglobulin G2 (IgG2)	5.33 g/L	2.418-7.003 g/L	Within normal range
Immunoglobulin G3 (IgG3)	2.70 g/L	0.2182-1.7606 g/L	Elevated
Immunoglobulin G4 (IgG4)	1.87 g/L	0.0392-0.864 g/L	Markedly elevated, supportive of IgG4-RD

An ultrasound of the cervical region identified multiple enlarged hypoechoic lymph nodes in the submandibular, supraclavicular, parotid, jugular, and posterior triangle regions, with features suggestive of reactive lymphadenopathy (Figure 1). Given her persistent symptoms, she underwent a positron emission tomography-computed tomography (PET-CT) scan, which revealed multiple enlarged fluorodeoxyglucose (FDG)-avid nodes in the sub-parotid glands bilaterally; multiple bilateral lymphadenopathies involving the cervical, axillary, mediastinal, retroperitoneal, and mesenteric lymph nodes; and diffuse mild-to-moderate hypermetabolism at the pancreas, most prominent in the head and neck region (Figure 2).

**Figure 1:**
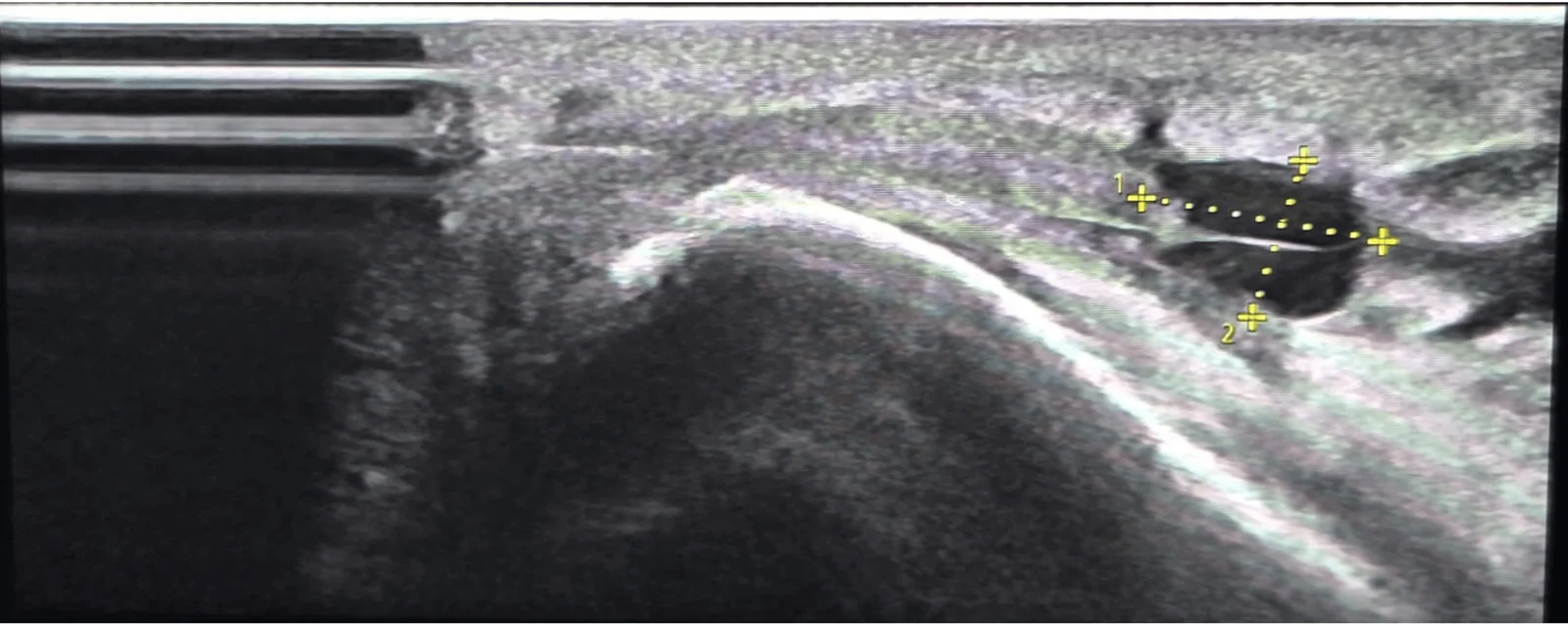
Ultrasound of the cervical region showing an enlarged hypoechoic lymph node (calipers 1 and 2) in the submandibular region, with features suggestive of reactive lymphadenopathy.

**Figure 2:**
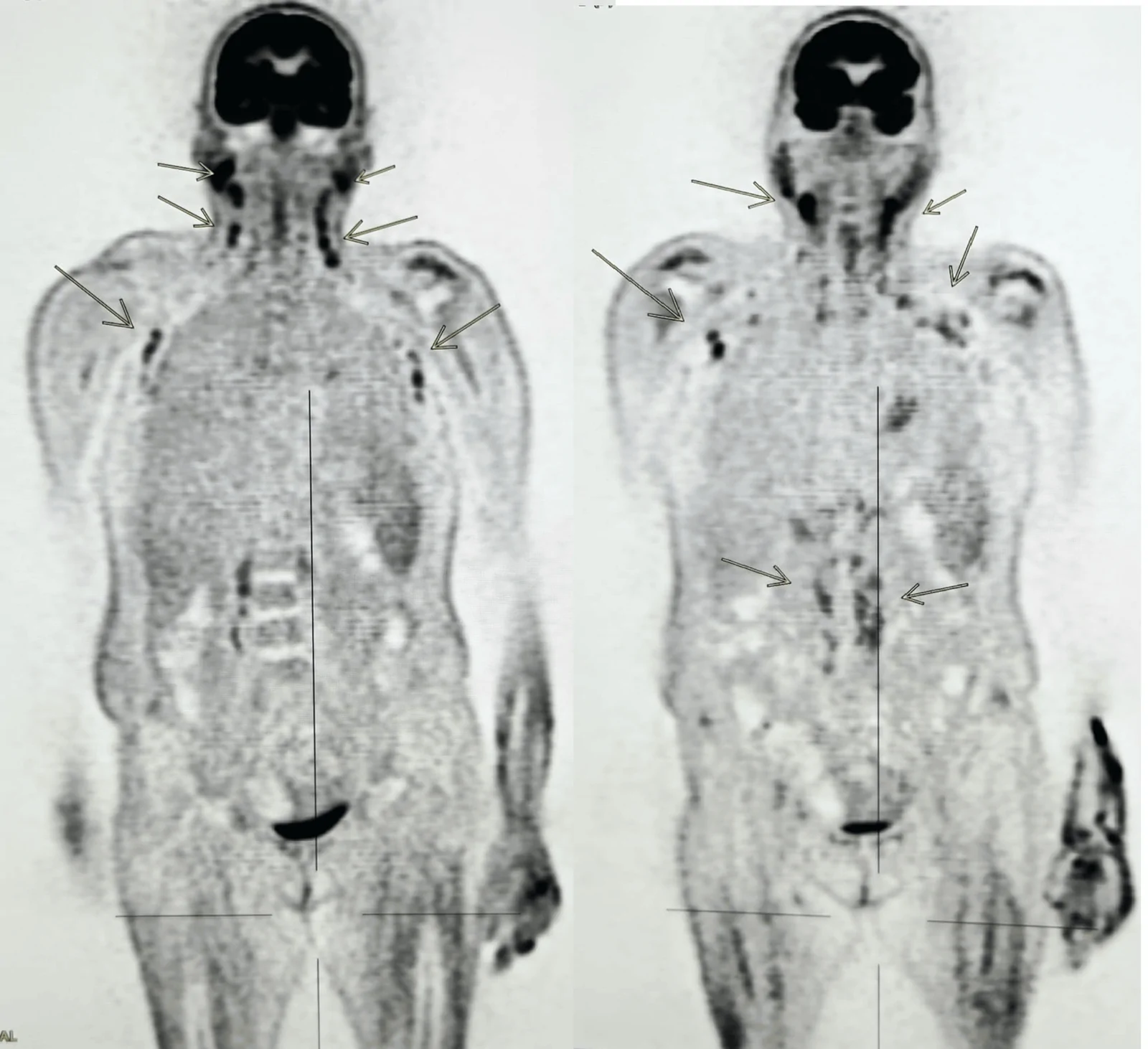
Positron emission tomography-computed tomography (PET-CT) scan, anterior whole-body views, showing multiple bilateral fluorodeoxyglucose (FDG)-avid lymph nodes (arrows) in the cervical, supraclavicular, axillary, mediastinal, retroperitoneal, and mesenteric regions.

A cervical lymph node excisional biopsy showed benign reactive plasma cell-rich follicular and sinus hyperplasia, with no evidence of granulomas, infectious agents, or hematolymphoid neoplasm. Immunohistochemistry revealed increased IgG4-positive plasma cells and polytypic kappa and lambda light chains (Figures 3, 4), arguing against a clonal lymphoproliferative disorder.

**Figure 3:**
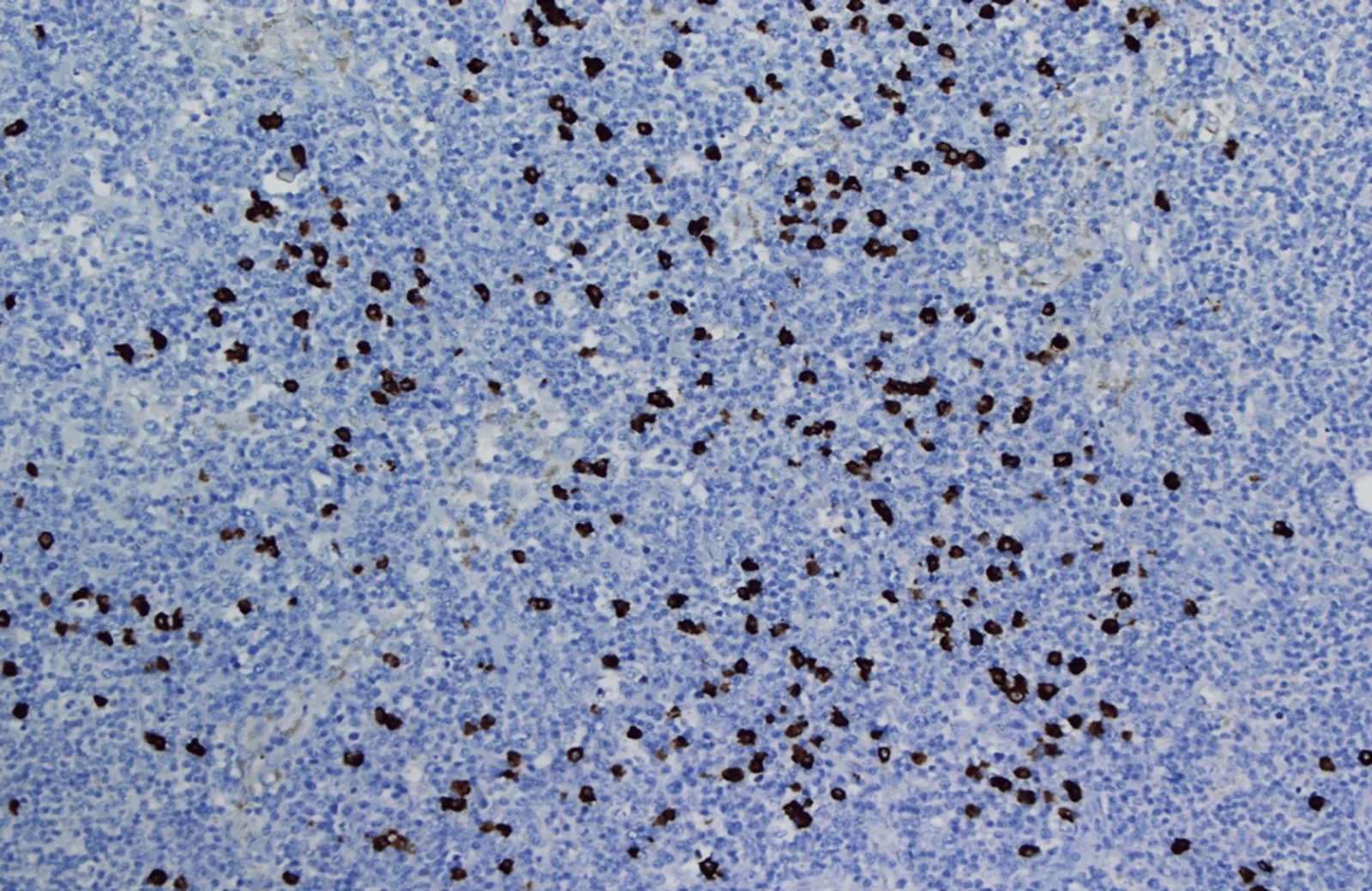
IgG4 immunomarker showing prominent IgG4-positive plasma cells, with 90 to 95 IgG4 plasma cells counted in one high-power field, representing 20% to 30% of the total IgG plasma cells.

**Figure 4:**
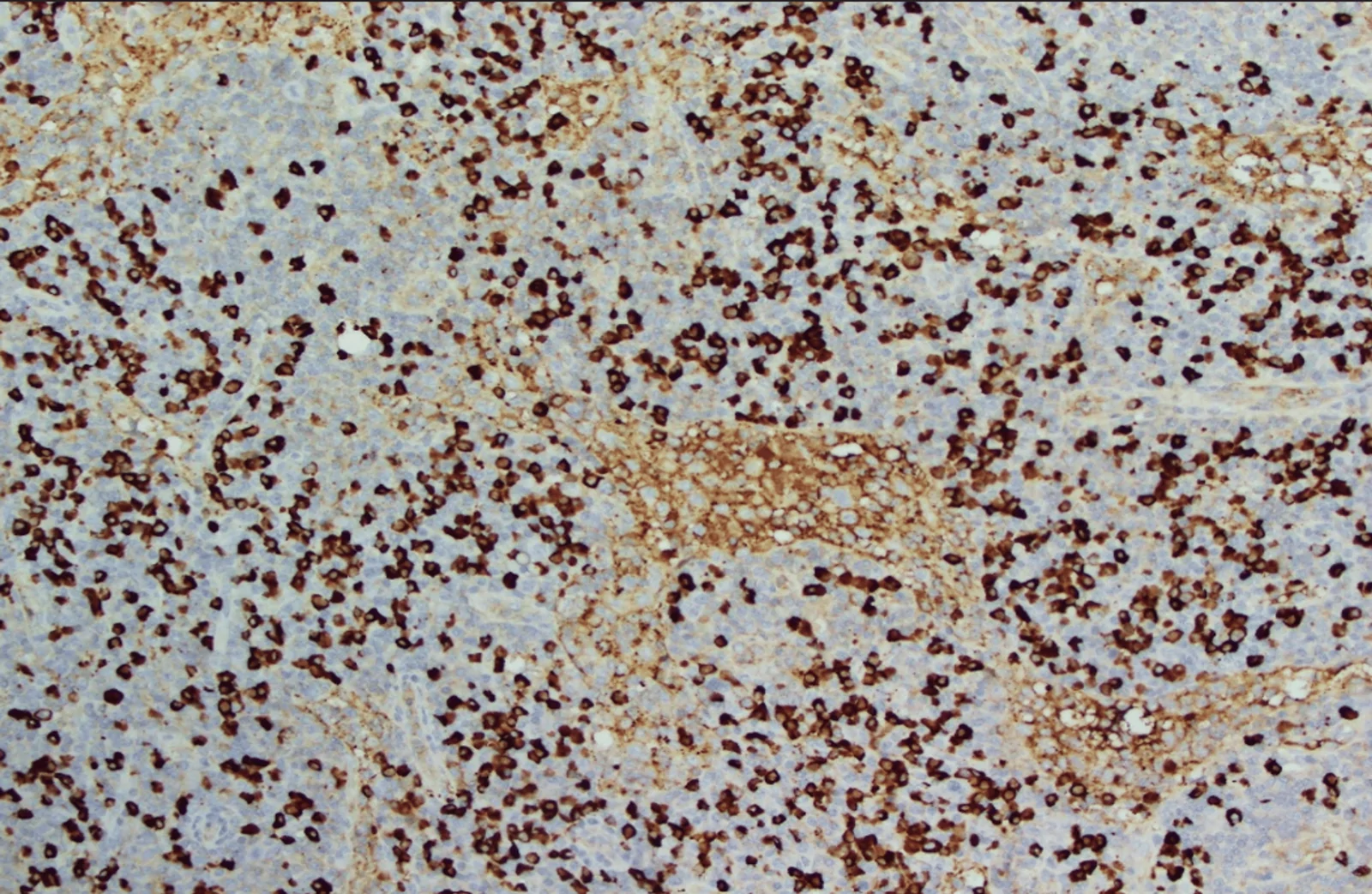
Kappa light chain immunomarker showing numerous plasma cells in equal proportion to lambda.

The rheumatology team initiated intravenous hydrocortisone 50 mg every 12 hours. She also received her first dose of intravenous rituximab 1000 mg, on which she improved greatly. She was discharged in stable condition with outpatient rheumatology follow-up and a plan to repeat CT imaging. The infectious disease team was also involved regarding her positive CMV and EBV IgM and IgG, which were later determined to be false positives.

## Discussion

IgG4-RD is characterized by mass-forming lesions, storiform fibrosis, and significant infiltration of lymphoplasmacytic cells rich in IgG4-positive plasma cells; these pathological features can resemble malignancies, infections, or other autoimmune diseases. The most commonly affected organs are the pancreas, lacrimal and salivary glands, retroperitoneum, orbit, lymph nodes, kidneys, and lungs, and the disease is often diagnosed incidentally through imaging or histopathological evaluation [10]. Lymphadenopathy is seen in up to 60% of cases [11].

The pathogenesis of IgG4-RD involves interaction between activated B lymphocytes and CD4+ cytotoxic T cells, which induce cytokine-mediated activation of fibroblasts and subsequent tissue fibrosis [10]. Some research has suggested that EBV might play a role in the development of IgG4-RD, particularly when the salivary glands are involved, although this association remains unclear [12]. In this case, EBV could have contributed to disease onset, or its presence might reflect prior immunosuppressive therapy rather than a direct causative role.

A notable hematologic hallmark of IgG4-RD is polyclonal hypergammaglobulinemia, often involving simultaneous elevations in multiple immunoglobulin subclasses, including IgG4 itself [13]. This is best explained by increased production of polyclonal antibodies by B cells and plasmablasts. These antibodies often show diverse, low-affinity reactivity, predisposing to nonspecific binding and cross-reactivity in serologic assays, including those for viral antigens and IgM responses, which can produce false-positive or nonspecific results in the setting of polyclonal hypergammaglobulinemia. This can mislead physicians into assuming an infectious or autoimmune etiology [13]. In this case, positive EBV and CMV IgM/IgG were initially thought to represent viral reactivation and were considered a possible contributor to the clinical picture; however, this interpretation became less convincing on further evaluation. EBV DNA PCR was detectable but below the quantification threshold, and tissue studies performed in this case did not demonstrate evidence of an EBV-driven process, arguing against clinically significant active EBV infection as the sole explanation. The serologic positivity is therefore better interpreted as a confounding feature, possibly related to nonspecific cross-reactivity and antigen-binding in the setting of polyclonal hypergammaglobulinemia in IgG4-RD.

According to the 2020 Revised Comprehensive Diagnostic Criteria for IgG4-Related Disease proposed by the Japanese IgG4 Research Group, a serum IgG4 concentration ≥1.35 g/L is a supportive criterion for diagnosis [7]. Nevertheless, this elevation is not exclusive to IgG4-RD, as mildly raised levels can occur in atopic, autoimmune, or infectious conditions. Thus, quantitative IgG4 measurement alone lacks specificity and must be interpreted in the broader clinical, radiologic, and histopathologic context.

Diagnosing IgG4-RD is a challenging process that requires integration of clinical, imaging, laboratory, and histopathological data. The 2019 American College of Rheumatology/European League Against Rheumatism (ACR/EULAR) classification criteria provide a framework to support diagnosis. While the criteria do not independently establish the diagnosis, they support disease classification through a three-step process: first, identifying involvement in at least one of 11 typical organs; second, ruling out alternative diagnoses using 32 exclusion criteria; and third, applying eight weighted inclusion domains spanning clinical, serological, imaging, and histopathological parameters. A cumulative score of 20 or above supports an IgG4-RD diagnosis, with a reported specificity of 97.8% and sensitivity of 82% [14]. In this case, the patient fulfills the entry criteria, with lymph node involvement showing plasma cell-rich follicular and sinus hyperplasia consistent with IgG4-RD, and none of the exclusion criteria apply. Based on the scoring system, she received 11 points for histopathologic findings (dense lymphocytic infiltrate with numerous IgG4-positive plasma cells), six points for organ involvement (multifocal lymphadenopathy), and 11 points for elevated serum IgG4 concentration, totaling 28 points. As this exceeds the threshold of 20, the patient meets the 2019 ACR/EULAR classification criteria for IgG4-RD.

Glucocorticoids remain first-line therapy for IgG4-RD. Current guidelines recommend oral prednisone at 0.6 mg/kg/day (30-40 mg/day) for induction over two to four weeks, followed by a gradual taper of 5 mg every one to two weeks; maintenance therapy is continued at 2.5-7.5 mg/day for at least six months in patients at risk of relapse [15]. In our patient, intravenous hydrocortisone (50 mg every 12 hours) was used initially instead of oral prednisone due to the acute systemic presentation and hospitalization, followed by a single 1000 mg dose of intravenous rituximab. This approach aligns with reported practices for severe, multisystem, or refractory IgG4-RD, where early combination therapy with glucocorticoids and rituximab can induce faster remission and reduce relapse rates [16]. Rituximab was used early in this case because of the multisystem involvement, disease severity, and underlying autoimmune comorbidities noted earlier in the patient's history. For patients who are intolerant to steroids or require steroid-sparing therapy, immunosuppressive agents or biologics such as rituximab may be used for induction and maintenance [16]. Prompt initiation of glucocorticoids, with tapering and maintenance as recommended, followed by rituximab when indicated, led to clinical improvement in this patient, underscoring the importance of early recognition and appropriate therapy in IgG4-RD.

## Conclusions

This case highlights the diagnostic complexity of IgG4-RD in a patient with systemic inflammation, generalized lymphadenopathy, autoimmune background, and overlapping viral serologies. The diagnosis was supported by the combined clinicopathologic picture, including elevated serum IgG4, plasma cell-rich lymph node histology with increased IgG4-positive plasma cells, compatible multisystem involvement, and clinical improvement after corticosteroids and rituximab.

The 2019 ACR/EULAR classification criteria provided a useful framework supporting the diagnosis in this case; however, these criteria were applied as a classification aid rather than a diagnostic tool. EBV/CMV seropositivity was an important confounder during the workup, but its exact relationship to disease onset remains uncertain. This case emphasizes the value of multidisciplinary assessment and careful clinicopathologic correlation in distinguishing IgG4-RD from infection, malignancy, and other inflammatory mimics.
